# Enzymatic Strategies for Biocontrolling Phytopathogenic Fungi Using *Trichoderma Koningiopsis* LBM116

**DOI:** 10.1111/1758-2229.70122

**Published:** 2025-06-12

**Authors:** Natalia Soledad Amerio, Marcela Paola Barengo, Gustavo Angel Bich, Pedro Dario Zapata, Laura Lidia Villalba, María Lorena Castrillo

**Affiliations:** ^1^ Universidad Nacional de Misiones. Facultad de Ciencias Exactas, Químicas y Naturales. Instituto de Biotecnología Misiones “Dra. María Ebe Reca”‐InBioMis. Laboratorio de Biotecnología Molecular Posadas Argentina; ^2^ Consejo Nacional de Investigaciones Científicas y Técnicas (CONICET) Posadas Argentina

**Keywords:** biological control, chitinases, enzymatic formulation, glucanases, proteases, sustainable agriculture

## Abstract

The growing demand for sustainable alternatives to chemical fungicides has driven the development of microbial‐based biocontrol strategies. In this study, the native strain *Trichoderma koningiopsis* LBM116 (Misiones, Argentina) was optimised for the production of mycolytic enzymes (chitinases, β‐1,3‐glucanases, and proteases) using factorial and response surface experimental designs. Enzyme secretion was increased by more than 250% compared to initial conditions by selecting specific carbon and nitrogen sources and adjusting inoculum and pH parameters. The optimised enzyme formulation improved lettuce seed germination to 86.66% in the presence of the phytopathogen *Fusarium* sp., under controlled conditions. In seedling trials, it also reduced disease severity and improved growth parameters. These results confirm the dual effect of the enzyme formulation, acting as a biocontrol agent and plant growth promoter. This work highlights the potential of enzyme formulations derived from *T. koningiopsis* LBM116 as an effective, low‐cost, and sustainable alternative for managing phytopathogens in agriculture.

## Graphical abstract

Optimised production of chitinase, β‐1,3‐glucanase, and protease by *Trichoderma koningiopsis* LBM116 led to an over 250% activity increase, and the enzymatic formulation was validated as an effective biocontrol agent against *Fusarium* sp. under controlled conditions.

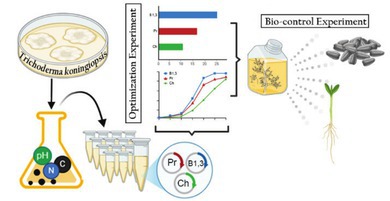

## 1 Introduction

Agriculture is the result of a set of human actions that transform the environment to give rise to products, food, or other goods whose main objective is to provide them to the growing population of each country, in addition to granting a satisfactory income for the farmers (Deguine et al. 2021). Modern agriculture is heavily dependent on technology and science. Vegetal sanitation and conservation are vital for thriving agriculture since yield and productivity of the crops are key points from the economic point of view and must take into account an optimal synergy of the control system pest–plant (Guo et al. 2023). The global trend is strongly directed towards using agrochemicals for controlling pests in crops due to their positive effect on the enhanced and fast elimination of pests (Kumar et al. 2019). However, the improper use of these products has resulted in significant adverse effects, such as water pollution, soil degradation, pesticide resistance, impacts on human health, loss of biodiversity, and air pollution (Vinale et al. 2008).

All these problems have led governments, farmers, and society to seek more environmentally friendly production alternatives, finding substitutes such as organic agriculture and biocontrol as more sustainable and ecological replacements (He et al. 2021; Gunjal 2023; Hossain et al. 2023). The latter has been growing worldwide in recent years. Using biological supplies based on beneficial microorganisms to protect crops and conserve natural resources is a possible solution to this problem (Ranasingha et al. 2024).

In the world market, there are different types of biocontrol agents depending on the group of pests, among which are the fungi of the genus *Trichoderma* (Gunjal 2023). They stand out for being widely studied and used due to their great effectiveness and good performance against phytopathogenic fungi. Several mechanisms have been suggested to play a role in controlling plant disease by *Trichoderma* isolates. It was reported that they can produce extracellular lytic enzymes such as chitinases (EC 3.2.1.14—EC 3.2.1.200), glucanases (EC 3.2.1.39—EC 3.2.1.58), and proteases (EC 3.4.21.63) (Kanauchi and Bamforth 2001; Sharma et al. 2022). This observation, together with the fact that chitin and β‐1,3‐glucan are the main structural components of the fungal cell wall, suggests that chitinases and β‐1,3‐glucanases produced by some *Trichoderma* isolates are key enzymes in the lysis of cell walls during mycoparasitic action, being most frequently considered to play an essential role in biocontrol. On the other hand, *Trichoderma* proteases have been reported to participate in the host lysis by attacking lipids and proteins, which are also part of the cell–wall skeleton (Viterbo et al. 2002).

A key area in the development process of a bioformulation based on beneficial microorganisms is combining biocontrol agents with enzymes or other components derived from microorganism metabolism (Fadiji et al. 2024). This combination would improve the effectiveness and speed up the bioformulation's action times. Although several fungicides based on *Trichoderma* formulations have been marketed worldwide in recent years, there is still considerable interest in finding and formulating more efficient products based on this fungus (da Silva Medina et al. 2024).

Successful enzyme production by filamentous fungi requires studying and optimising the effects of different nutritional and incubation factors (Rousta et al. 2021; Alharbi et al. 2023). Therefore, this work aimed to optimise a liquid culture medium to enhance extracellular enzyme secretion by *T. koningiopsis* LBM116 and validate its efficacy through in vivo assessments. This optimisation aims to develop an effective enzymatic formulation in the biological control of phytopathogenic fungi affecting crops.

## 2 Materials and Methods

### 2.1 Fungal Strains and Maintenance

The *Trichoderma* strain from native soils of the province of Misiones, Argentina (*T. koningiopsis*), used in this research, was selected based on its high antagonistic capacity evaluated in vitro previously (Castrillo et al. 2013; Castrillo et al. 2014; Castrillo et al. 2017; Castrillo et al. 2021; Castrillo et al. 2023). The strain was deposited in the Culture Collection of the Laboratory of Molecular Biotechnology (in Spanish, Laboratorio de Biotecnología Molecular) of the Institute of Biotechnology Misiones (National University of Misiones) under accession code LBM 116. The microbial culture was grown and maintained on potato dextrose agar (PDA) at 4°C.

### 2.2 Selection of Carbon and Nitrogen Sources for Enzyme Secretion *by T. Koningiopsis* LBM116

A combination of different carbon and nitrogen sources was used: Colloidal chitin (*QC*) 0.18% (^w^/_v_), gelatin (*Gel*) 5% (^w^/_v_), cell walls of *Fusarium* sp. treated (*Fit I*) 2 g L^−1^ and cell walls of *Fusarium* sp. untreated (*Fit II*) 0.2% (^v^/_v_) (Table 1), as carbon sources were used. A minimal medium (2 g L^−1^ KH_2_PO_4_; 0.4 g L^−1^ CaCl_2_.2H_2_O; 0.3 g L^−1^ MgSO_4_.4H_2_O; 0.005 g L^−1^ FeSO_4_.7H_2_O; 0.002 g L^−1^ MnSO_4_.4H_2_O; 0.002 g L^−1^ ZnSO_4_.7H_2_O; 0.003 g L^−1^ CoCl_2_.6H_2_O) supplemented with different nitrogen sources: 0.3 g L^−1^ urea (*Ur*); 1.4 g L^−1^ ammonium sulfate (*Sa*), 0.25 g L^−1^ yeast extract (*Ex*) and Mandels' solution (*Ma*) (Mandels and Reese 1960) were evaluated. All Erlenmeyer flasks were autoclaved for 15 min at 121°C and 1 psi pressure. *T. koningiopsis* LBM116 was inoculated with 2.5 mL of a conidia suspension with a final concentration of 1 × 10^7^ conidia mL^−1^ in 250 mL Erlenmeyer flasks, containing 60 mL of each medium. The inoculated Erlenmeyer flasks were incubated in the dark at 28°C ± 1°C at 100 rpm for 8 days. An aliquot of 3 mL of the culture supernatant was extracted every 48 h from each experiment to determine chitinase, β‐1.3‐glucanase, and protease activities.

**TABLE 1:** Enzymatic secretion by *T. koningiopsis* LBM116 in combination with different carbon and nitrogen sources at day 8.

Carbon source/Nitrogen source	Enzymatic activity		
	Chitinase U L^−1^	β‐1.3‐glucanase U L^−1^	Protease mg L^−1^
QC‐Ma	21.5 ± 1.26 ^g^	301.04 ± 0.38 ^j^	0 ^a^
QC‐Ur	12.9 ± 0 ^abc^	164.45 ± 0.78 ^h^	0 ^a^
QC‐SA	16.9 ± 0.31 ^ef^	109.84 ± 2.31 ^g^	0 ^a^
QC‐Ex	21,9 ± 1.98 ^fg^	394.54 ± 3.45 ^k^	0 ^a^
Gel‐Ma	10.5 ± 0.14 ª	58.5 ± 0.76 ^d^	102.02 ± 2.58 ^f^
Gel –Ur	12.52 ± 0.31 ª^bc^	48.72 ± 1.52 ^c^	27.61 ± 0.83 ^b^
Gel –SA	10.35 ± 0.04 ª	67.65 ± 0.77 ^e^	36.46 ± 1.66 ^c^
Gel –Ex	11.98 ± 0.35 ª^b^	70.35 ± 0.76 ^e^	119.07 ± 1.66 ^gh^
Fit I‐Ma	**29.1 ± 1.88** ^**h**^	**590.3 ± 4.90** ^**n**^	296.3 ± 2.5 ^j^
Fit I‐Ur	15.30 ± 3.22 ^cde^	454.65 ± 0.71 ^m^	121.43 ± 5 ^h^
Fit I‐SA	16.05 ± 4.05 ^de^	442.89 ± 0.19 ^L^	74.82 ± 2.5 ^e^
Fit I‐Ex	**28,95 ± 0.04** ^**h**^	**591.65 ± 3.07** ^**n**^	**391.80 ± 1.75** ^**k**^
Fit II‐Ma	14.3 ± 0.13^bcde^	36,0 ± 0.38 ^b^	190 ± 0.52 ^i^
Fit II‐Ur	10.90 ± 0.09 ^a^	15,14 ± 0.01 ª	116.71 ± 1.66 ^g^
Fit II‐SA	12.93 ± 0.18 ^abcd^	240,90 ± 1.28 ^i^	0 ^a^
Fit II‐Ex	10.67 ± 0.04 ^a^	93,55 ± 3.05 ^f^	61.25 ± 1.68 ^d^

*Note:* The different letters in the columns represent statistically significant differences (*p* < 0.05). The highest values related to enzymatic secretion are highlighted in bold.

### 2.3 Cell Wall Preparation

The cell wall of *Fusarium* sp. was prepared using the method modified by Cortes et al. (1998). Erlenmeyer flasks (500 mL) containing 100 mL of glucose 0.5% (^w^/_v_) and malt extract 1.27% (^w^/_v_), as a carbon source and *Ma* as a nitrogen source, were incubated with four discs of 5 mm^2^ of an actively growing mycelium of *Fusarium* sp. grown on PDA and incubated at 28°C for 14 days. The mycelium was then collected by filtration through Whatman no. 1 filter paper, washed with distilled water, and allowed to stand in 0.85% 2 M NaCl for 2 h. Then heated in 2% SDS for 5 min and centrifuged (4500×g) for 10 min. The collected mycelium was washed with a chloroform: methanol solution (1:1) and centrifuged again (4500×g) for 10 min. Finally, a final wash was performed with acetone and subsequent centrifugation (4500×g) for 10 min. The mycelium was allowed to dry at room temperature for 24 to 48 h, crushed in a mortar, and stored in the freezer until use.

### 2.4 Enzyme Activity Assays

Chitinase activity was assayed using the method described by Wen et al. (2002). One unit (U) of chitinase activity was defined as the amount of enzyme necessary to release 1 μmol of NAG per minute.

The β‐1.3‐glucanase activity was assayed by the method described by Masih and Paul (2002). One unit (U) of β‐1.3‐glucanase activity was defined as the amount of enzyme necessary to release 1 μmol of glucose/min/mL.

The protease activity was assayed by the method described by Charney and Tomarelli (1947) with modifications. The enzymatic reaction mixture containing 300 μL of the enzyme samples and 300 μL of azocasein 0.5% (^w^/_v_) dissolved in 0.2 M tris–HCl buffer (pH 7.4) was incubated for 50 min at 37°C. The amount of p‐nitrophenol released was measured at 410 nm after adding 10% Na_2_CO_3_ to the reaction mixtures. The reaction was terminated by 600 μL of trichloroacetic acid 10% (^w^/_v_) and centrifuged at 7000 g for 10 min to decant the undegraded substrate. 800 μL of the supernatant was extracted with 800 μL of NaOH (1 M). The enzyme activity was calculated as the amount of enzyme needed to increase the absorbance at 440 nm by 0.01 units under the assay conditions.

### 2.5 Effect of Different Concentrations of Carbon Source and Varied Types of Nitrogen Sources on Enzyme Secretion *by T. Koningiopsis* LBM116

The two previously selected nitrogen sources, with known capacity for increasing chitinase production, β‐1.3‐glucanase, and protease, were evaluated with different concentrations of the selected carbon source. A multilevel factorial design was carried out in 16 experimental runs. The nitrogen sources were screened at two levels (−1 *Ex* and + 1 *Ma*). The carbon sources were screened at four levels (+1 = 3 g L^−1^; +0.33 = 2 g L^−1^; −0.33 = 1 g L^−1^; −1 = 0.5 g L^−1^). The culture media were inoculated as previously described and incubated at 28°C ± 1°C with shaking at 100 rpm for 10 days. An aliquot of 3 mL of the culture supernatant was extracted every 48 h from each experiment to determine chitinase, β‐1.3‐glucanase, and protease activities.

### 2.6 Optimization of Carbon Source Concentrations on Enzyme Secretion by *T. Koningiopsis* LBM116

To enhance the chitinase, β‐1.3‐glucanase, and protease secretion by *T. koningiopsis* LBM116, the effects of new concentrations of the carbon source (*Fit I*) in combination with the selected nitrogen source (*Ex*) were tested. A factorial design was carried out. The carbon sources were screened at four levels (+1 = 3 g L^−1^; +0.33 = 5 g L^−1^; −0.33 = 7 g L^−1^; −1 = 9 g L^−1^). The culture media were inoculated as previously described and incubated at 28°C ± 1°C with shaking at 100 rpm for 14 days. An aliquot of 3 mL of the culture supernatant was extracted every 48 h from each experiment to determine chitinase, β‐1.3‐glucanase, and protease activities.

### 2.7 Optimization of Nitrogen Source Concentrations, Inoculum Concentration, and Culture Medium pH on Enzyme Secretion by *T. Koningiopsis* LBM116

A response surface design (RSM) called Box Behnken (1960) was carried out to test the effect of each of the factors at three levels (high, low, and medium). The factors concentration of Ex as a nitrogen source (+1 = 0.2 g L^−1^; 0 = 1.1 g L^−1^; −1 = 2 g L^−1^); initial inoculum concentration (+1 = 2 × 10^7^; 0 = 1 × 10^7^; −1 = 1 × 10^6^) and initial pH of the culture medium (+1 = pH 7; 0 = pH 5.5; −1 = pH 4) were analysed. The design consisted of 17 experimental runs that included a quintuplicate central point. This central point is additional tests located at a midpoint between the low and high levels of all the factors, which allows a more uniform estimation of the variance in the entire design space. The culture media were inoculated as previously described and incubated at 28°C ± 1°C with shaking at 100 rpm for 12 days. An aliquot of 3 mL of the culture supernatant was extracted every 48 h from each experiment to determine chitinase, β‐1.3‐glucanase, and protease activities.

To validate the model, six experiments were conducted using the optimised variables predicted by the RSM.

### 2.8 Bio‐Control Experiment

Bioassays were conducted to analyse the effect of the optimised enzyme formulation of *T. koningiopsis* LBM116 against the phytopathogen *Fusarium* sp. in lettuce ( *Lactuca sativa* Var. Capitata) seeds and seedlings. Both experiments used a completely randomised design in a germination chamber with 30% ± 2 humidity, 12:12 light, and 24°C ± 2.

First, the effect of biocontrol on seed germination without visible damage in the presence of the pathogen *Fusarium* sp. was evaluated using sterile sand‐filled aluminium trays as a substrate. The seeds were disinfected according to the method described by Valery and Reyes (2013), and conidia suspensions of *Fusarium* sp. (1 × 10^6^) and *T. koningiopsis* LBM116 (1 × 10^7^) were prepared to inoculate the substrate and treat the seeds, respectively. Four treatments were applied: disease control (untreated disinfected seeds exposed to the pathogen), absolute control (untreated disinfected seeds in sterile sand), LBM116 control (seeds treated with *T. koningiopsis* in sand containing the pathogen), and the enzyme formulation test (seeds treated with the enzyme formulation in sand containing the pathogen). Twenty seeds were placed in each tray, covered with perforated film for aeration, and kept in the dark. Each treatment was performed in triplicate. Seed germination percentages were assessed after 7 days. Germination percentages for each treatment were determined using the following formula: Germination Percentage = (Number of germinated seeds/Total number of seeds sown) × 100.

Secondly, the effect of biocontrol on lettuce seedlings in the presence of *Fusarium* sp. was evaluated. Seedlings, 7 days after germination, were transplanted to trays containing commercial substrate inoculated with the pathogen at a concentration of 3.6 × 10^6^ conidia/mL. The treatments consisted of a diseased control (seedlings exposed to the pathogen), an absolute control (seedlings without pathogen), a LBM116 control (seedlings treated with *T. koningiopsis* LBM116 in the presence of the pathogen), and a treatment with the enzyme formulation (seedlings treated with the optimised enzyme formulation in the presence of the pathogen). Trays with 10 seedlings each were used per treatment, each performed in triplicate. The enzyme formulation and the conidial suspension of *T. koningiopsis* LBM116 were applied once, 10 days after transplanting, by spraying 1 mL per seedling. After 30 days, seedlings were manually harvested and washed under running water to measure height (H), fresh and dry biomass of the aerial part (FBAP, DBAP), fresh root biomass (FRB), dry root biomass (DRB), and overall plant health status according to a graded scale (Deguine et al. 2021; Guo et al. 2023; Kumar et al. 2019; Vinale et al. 2008) by Scott et al. (2010): 0 = no symptoms, 1 = mild stunting, 2 = moderate stunting, 3 = severe stunting, 4 = dead or nearly dead plant. The disease severity index (DSI) was calculated for each treatment using the following formula: [((number of plants in class 1) + 2 (number of plants in class 2) + 3 (number of plants in class 3) + 4 (number of plants in class 4))/total number of plants] × 100/4.

### 2.9 Statistical Analyses

Statistical analysis was performed using Statgraphics Centurion XVI. I (StatPoint Inc. version 15.2.05) with a confidence level of 95% to analyse the significant parameters of the enzymatic activities. We expressed the quality of the fit to the polynomial model equation using the coefficient of determination R^2^, and we verified the significance of the regression coefficients using the *F*‐test and the *p*‐value. The R^2^ coefficient was calculated as an indicator of model fit. The model would be more robust, and the prediction of the response would be better if the R^2^ coefficient approaches. Data from the experiments were subjected to ANOVA and expressed as means with standard errors (SE). Treatment effects were determined using Duncan's multiple range test, and significances were expressed at *p* < 0.05. Graphics were generated using InfoStat Professional 2020 (Di Rienzo et al. 2020).

## 3 Results

### 3.1 Selection of Carbon and Nitrogen Sources for Enzyme Secretion

**FIGURE 1:**
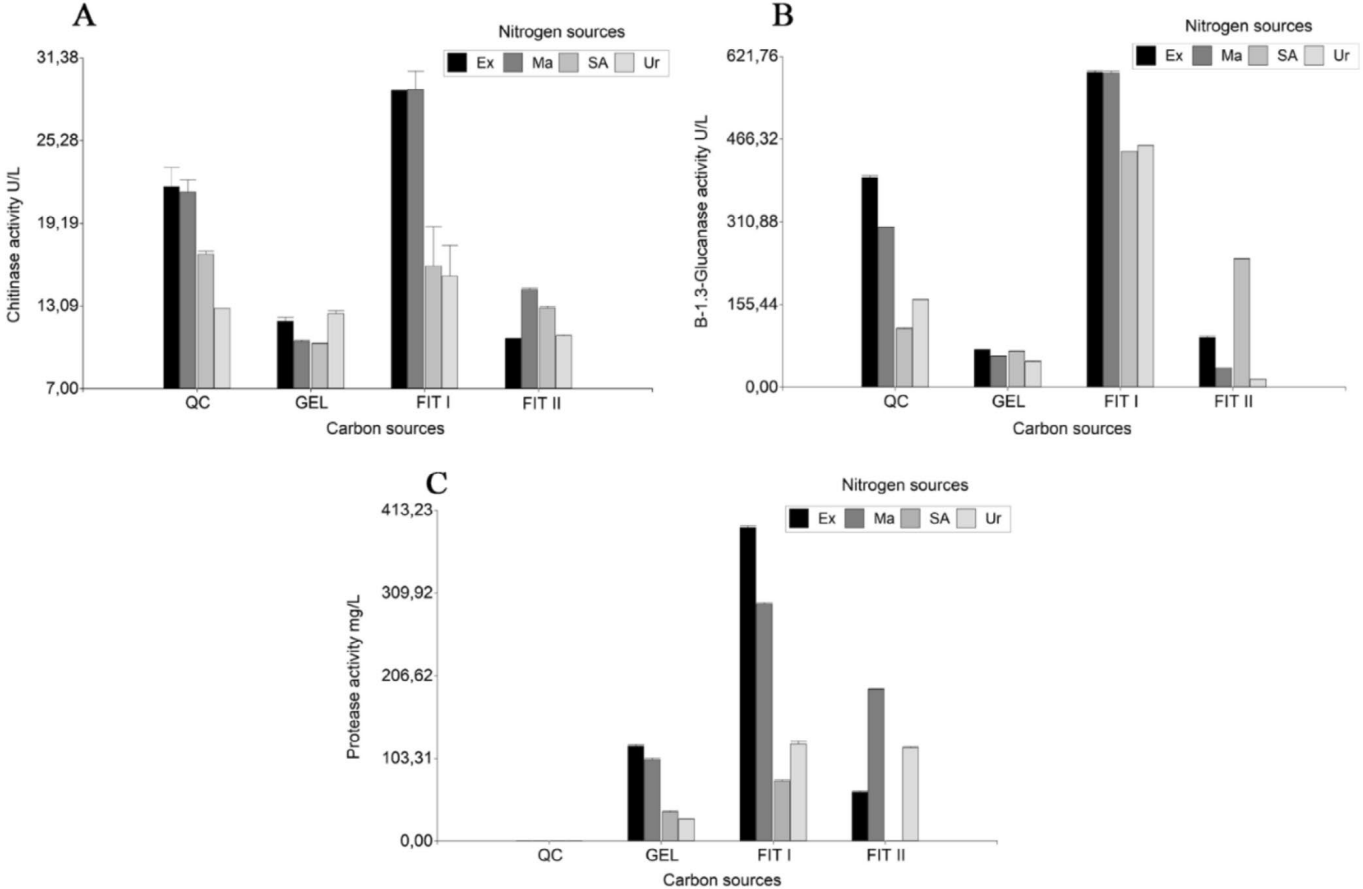
Enzymatic activity of *T. koningiopsis* LBM116 under different culture conditions. (A) Chitinase, (B) β‐1,3‐glucanase, and (C) Protease activities were measured in culture supernatants obtained after 8 days of growth. Carbon sources: QC: colloidal chitin; Gel: gelatin; Fit I: cell walls of *Fusarium* sp. treated; Fit II: cell walls of *Fusarium* sp. untreated. Nitrogen sources: Ex: yeast extract; Ma: mandels; SA: ammonium sulfate; Ur: urea. Bars represent mean values of three replicates ± standard error (SE).

The effects of *QC*, *Gel*, *Fit I*, and *Fit II*, in combination with various nitrogen sources, were evaluated for chitinase, β‐1.3‐glucanase, and protease secretion by *T. koningiopsis* LBM116 (Table 1). In combination with Ma and Ex, Fit I showed the highest enzyme activity (*p* ≤ 0.05) of the three tested enzymes. *T. koningiopsis* LBM116 secreted 29.1 ± 1.88 U L^−1^ and 28.95 ± 0.04 U L^−1^ of chitinase when it was grown with *Fit I* in combination with *Ma* or *Ex*, respectively, without statistically significant differences between them (*p* ≥ 0.05) (Figure 1A). *T. koningiopsis* LBM116 secreted 590.3 ± 4.90 U L^−1^ and 591.65 ± 3.07 U L^−1^ of β‐1.3‐glucanase when it was grown with *Fit I* in combination with *Ma* or *Ex*, respectively, without statistically significant differences between them (*p* ≥ 0.05) (Figure 1B). Finally, *T. koningiopsis* LBM116 secreted 391.80 ± 1.75 mg L^−1^ of protease when it was grown with *Fit I* in combination with *Ex*, presenting statistically significant differences between the other tests (*p* ≤ 0.05) (Figure 1C). These results were obtained on day 8 of incubation, showing statistically significant differences (*p* ≤ 0.05) with the other days tested. The other trials presented lower levels of enzymatic activity under the conditions studied.

As *Fit I* presented the highest chitinase, β‐1.3‐glucanase, and protease levels (*p* ≤ 0.05), the next experiments were conducted to optimise their concentrations. The nitrogen source continued to be optimised.

### 3.2 Effect of Different Carbon Source Concentrations and Different Types of Nitrogen Sources on Enzyme Secretion

**FIGURE 2:**
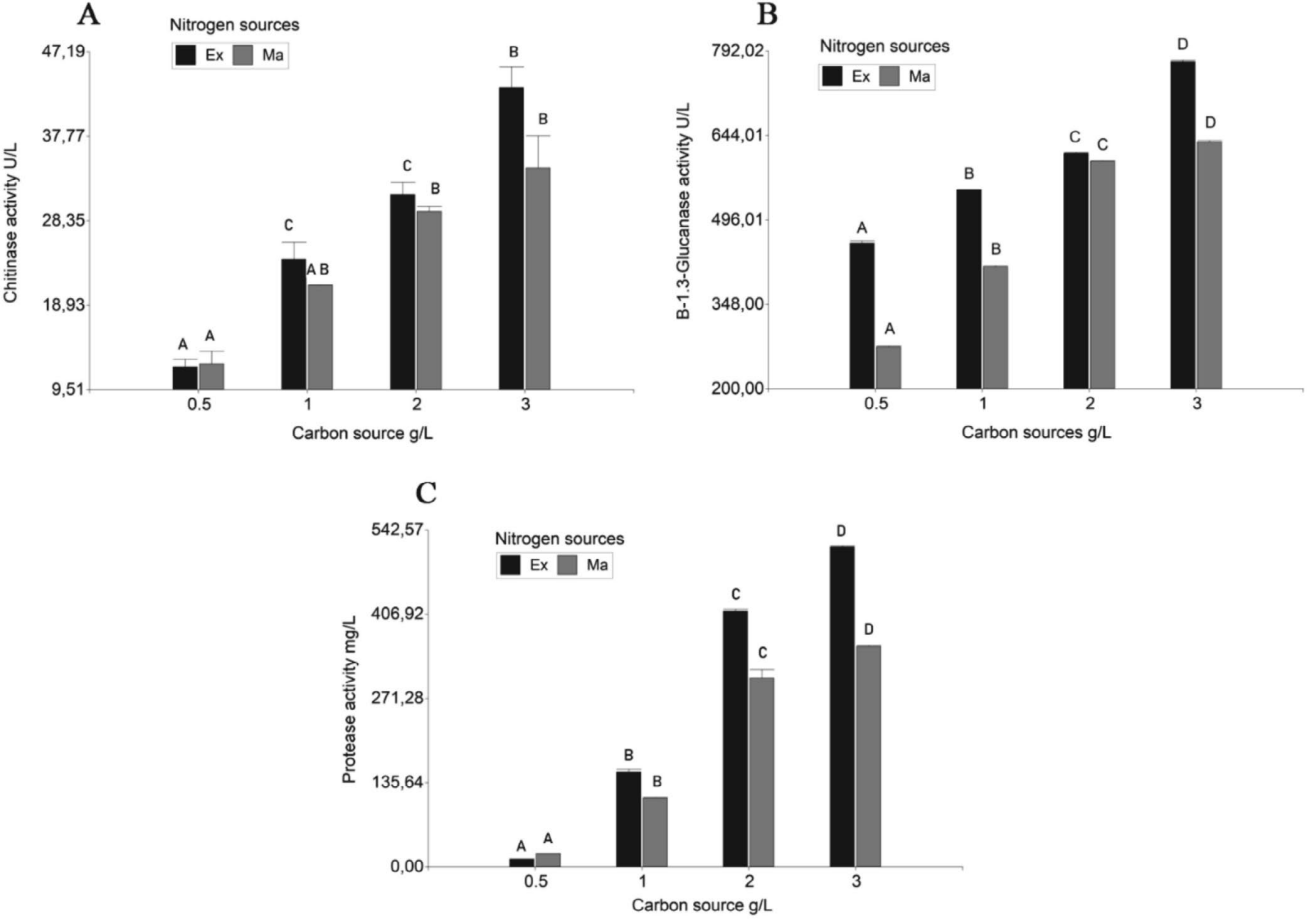
Effect of different concentrations of the carbon source *Fit I* combined with two nitrogen sources on enzyme secretion by Trichoderma koningiopsis LBM116 after 10 days of incubation. (A) Chitinase activity. (B) β‐1,3‐glucanase activity. (C) Protease activity. Bars represent mean values of three replicates ± standard error (SE). The different letters in the columns represent significant differences (*p* < 0.05).

*T. koningiopsis* LBM116 presented the highest chitinase, β‐1.3‐glucanase, and protease activity levels (*p* ≤ 0.05) when it was grown with 3 g L^−1^ of *Fit I* as a carbon source and *Ex* as a nitrogen source (Table 2). *T. koningiopsis* LBM116 secreted 43.18 ± 3.18 U L^−1^ of chitinase (Figure 2A). On the other hand, it secreted 773.54 ± 3.26 U L^−1^ of β‐1.3‐glucanase (Figure 2B) and 515.55 ± 2.50 mg L^−1^ of protease (Figure 2C). These results were obtained on day 10 of incubation, showing statistically significant differences (*p* ≤ 0.05) with the other days tested. The multiple regression analysis of this model showed R^2^ of 0.91, 0.97, and 0.96 for chitinase, β‐1.3‐glucanase, and protease, respectively. Moreover, the lack of fit was not significant in this model, indicating that the model adequately represented the observed data at a 95% confidence level. As *Ex* presented higher chitinase, β‐1.3‐glucanase, and protease levels (*p* ≤ 0.05), the next experiments were carried out with *Ex* as the nitrogen source, and different concentrations of *Fit I* continued to be optimised.

**TABLE 2:** A multilevel factorial design matrix for four carbon and two nitrogen sources with coded values (experimental values in parentheses) for chitinase, β‐1.3‐glucanase and protease activity levels of *T. koningiopsis* LBM116 at day 10.

Run no.	Carbon source g L^−1^	Nitrogen sources	Enzymatic activity		
			Chitinase U L^−1^	β‐1.3‐glucanase U L^−1^	Protease mg L^−1^
1	−1(0.5)	1(*Ma*)	11.02	274.93	21.12
2	1(3)	1(*Ma*)	37.80	634.59	356.26
3	1(3)	−1(*Ex*)	**45.47**	**771.22**	**513.78**
4	−1(0.5)	−1(*Ex*)	11.22	452.44	12.86
5	0.33(2)	1(*Ma*)	29.96	597.94	220.55
6	−0.33(1)	1(*Ma*)	21.07	415.32	109.36
7	−0.33(1)	−1(*Ex*)	25.95	549.16	156.88
8	0.33(2)	−1(*Ex*)	32.60	611.05	384.64
9	0.33(2)	−1(*Ex*)	29.97	614.06	389.18
10	1(3)	−1(*Ex*)	40.89	775.85	517.32
11	−1(0.5)	1(*Ma*)	13.78	272.92	20.5
12	0.33(2)	1(*Ma*)	28.84	597.94	221.73
13	−0.33(1)	−1(*Ex*)	22.14	549.16	148.44
14	−0.33(1)	1(*Ma*)	21.20	415.32	111.3
15	−1(0.5)	−1(*Ex*)	12.93	452.44	12.5
16	1z(3)	1(*Ma*)	30.72	634.59	353.24

*Note:* The highest values related to enzymatic secretion are highlighted in bold.

### 3.3 Optimization of Carbon Source Concentrations on Enzyme Secretion by *T. Koningiopsis* LBM116

**FIGURE 3:**
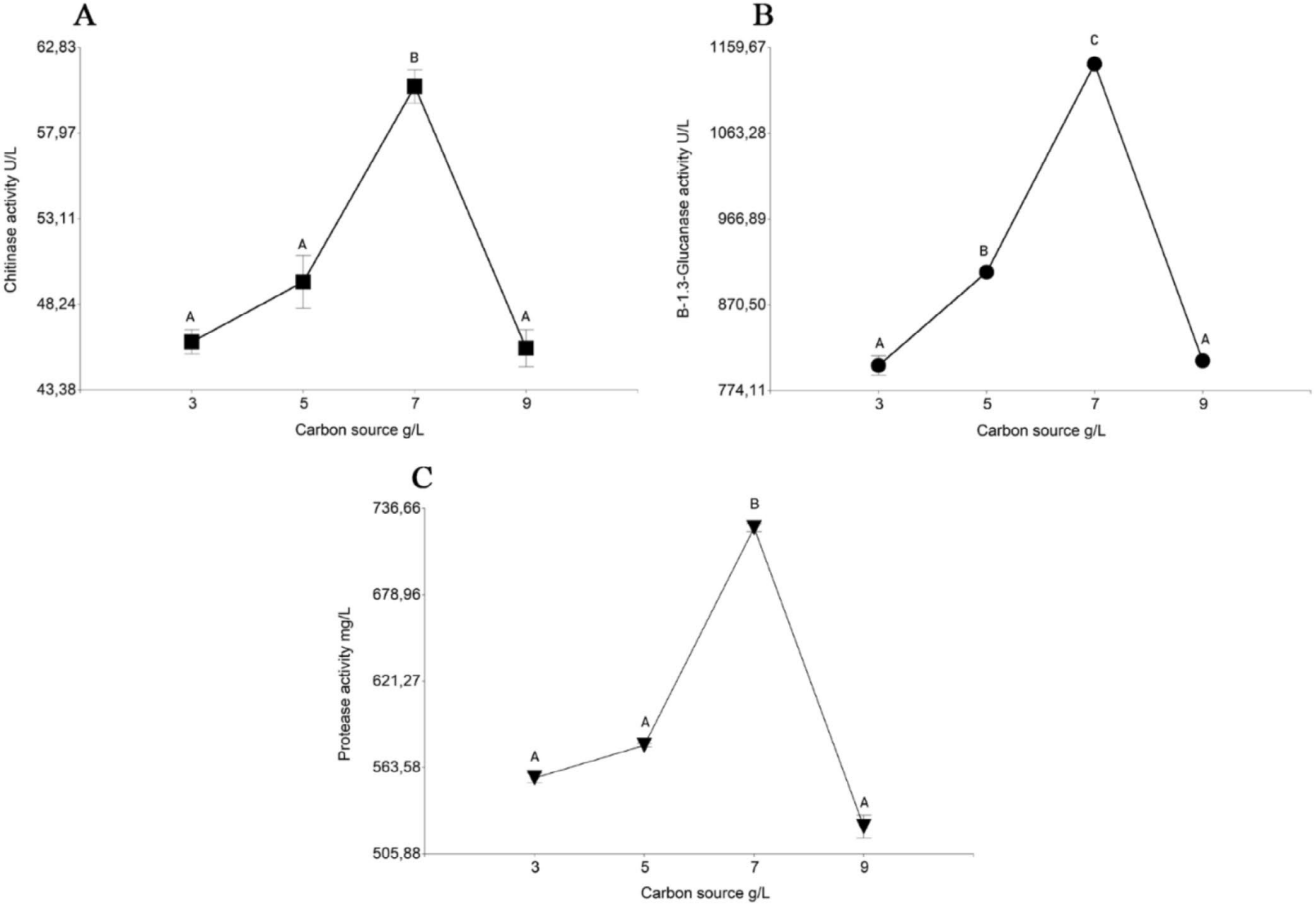
Effect of different concentrations of the carbon source *Fit I* on the secretion of enzymes by *T. koningiopsis* LBM116. (A) Chitinase activity, (B) β‐1,3‐glucanase activity, and (C) Protease activity. Bars represent mean values of three replicates ± standard error (SE). Different lowercase letters above the bars indicate statistically significant differences among treatments according to Tukey's test (*p* < 0.05).

Since the optimal concentration of the carbon source was the highest tested, we tried higher concentrations to optimise further, along with Ex, previously selected as a nitrogen source. *Fit I* 7 g L^−1^ had statistically significant effects (*p* ≤ 0.05) on chitinase, β‐1.3‐glucanase, and protease activity. *T. koningiopsis* LBM116 secreted 60.61 ± 1.31 U L^−1^ of chitinase (Figure 3A), 1141.21 ± 1.31 U L^−1^ of β‐1.3‐glucanase (Figure 3B), and 723.76 ± 3.40 mg L^−1^ of protease (Figure 3C). These results were obtained on day 12 of incubation, showing statistically significant differences (*p* ≤ 0.05) with the other days tested.

### 3.4 Optimization of Nitrogen Source Concentrations, Inoculum Concentration, and Culture Medium pH on Enzyme Secretion by *T. Koningiopsis* LBM116

**FIGURE 4:**
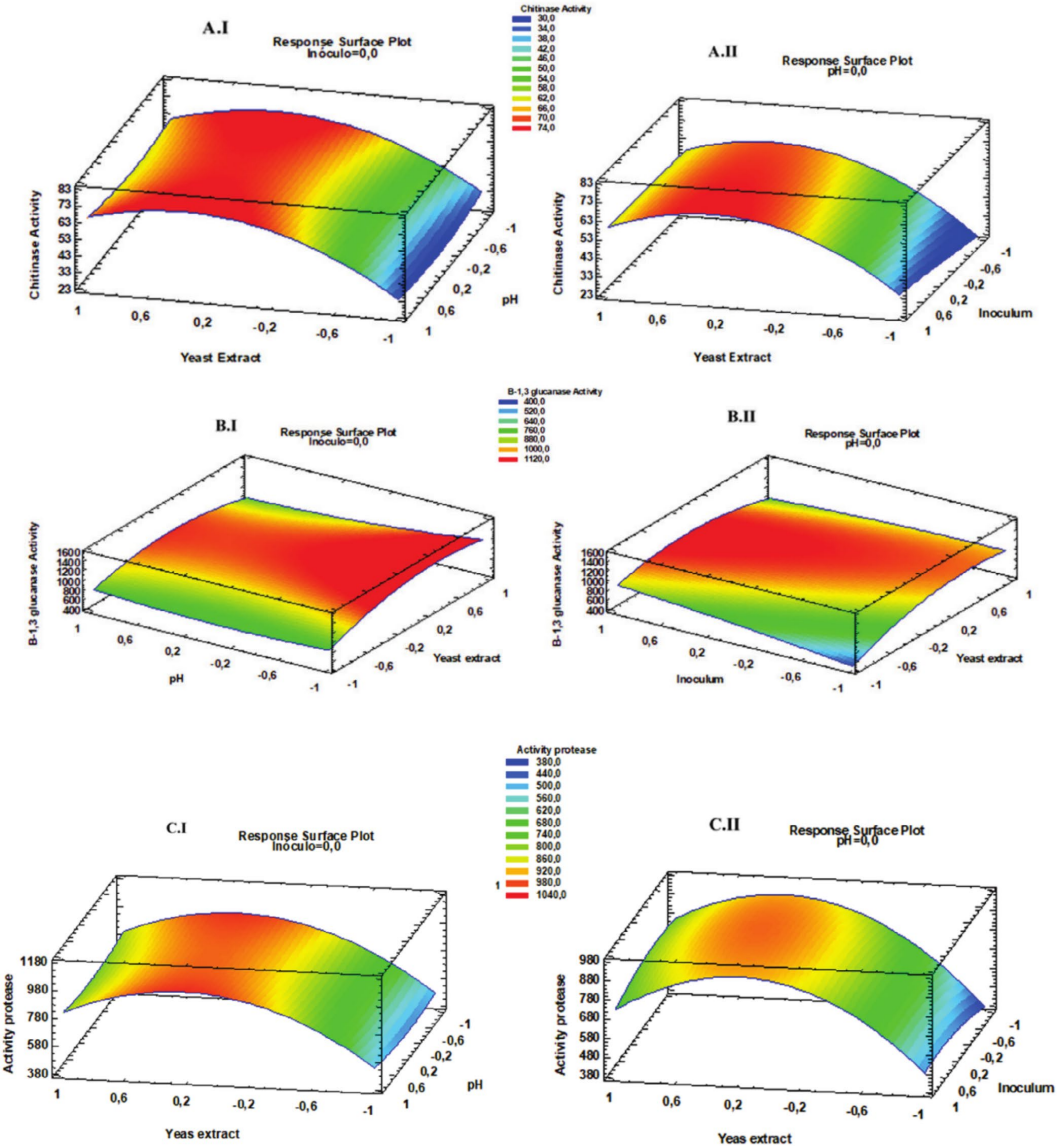
Three‐dimensional response surface plots for enzymatic secretion by *T. koningiopsis* LBM116. The plots show the interactive effects of *Ex* concentration, initial inoculum, and pH culture medium. (A) Chitinase activity I: *Ex* and pH when inoculum is fixed at its middle level; II: *Ex* and inoculum when pH is fixed at its middle level. (B) β‐1,3‐glucanase activity I: *Ex* and pH when inoculum is fixed at its middle level; II: *Ex* and inoculum when pH is fixed at its middle level. (C) Protease activity I: *Ex* and pH when inoculum is fixed at its middle level; II: *Ex* and inoculum when pH is fixed at its middle level.

The adequacy and significance of the regression models obtained from the Box–Behnken design were evaluated through ANOVA analyses. For all enzymatic activities (chitinase, β‐1,3‐glucanase, and protease), the models were statistically significant (*p* < 0.05), and the coefficients of determination (R^2^) were 96.73%, 92.50%, and 98.63%, respectively. Furthermore, the Lack of Fit tests were not significant (*p* > 0.05), confirming that the models adequately fit the experimental data. Detailed ANOVA results are presented in Tables 3, 4, 5. The chitinase activity was increased when the concentration of *Ex* was around its central point. The inoculum concentration was at the +1 level, and the pH was at the −1 level (Figure 4A). This model predicted an optimal chitinase activity of 75,66 U L^−1^ under the following conditions: 0.88 g L^−1^ *Ex*, inoculated with 2 × 10^7^ conidia mL^−1^ and pH 4. The β‐1.3‐glucanase activity was increased when the concentration of *Ex* was around its central point. The inoculum concentration was at the +1 level and the pH was at the −1 level (Figure 4B). This model predicted an optimal β‐1.3‐glucanase activity of 145,224 U L^−1^ under the following conditions: 1 g L^−1^ *Ex*, inoculated with 2 × 10^7^ conidia mL^−1^ and pH 4. Protease activity increased when the concentrations of Ex and inoculum were near their central points, and the pH was at the +1 level (Figure 4C). This model predicted an optimal protease activity of 994,502 mg L^−1^ under the following conditions: 0.88 g L^−1^ *Ex*, inoculated with 1.2 × 10^7^ conidia mL^−1^ and pH 4. Through an optimization analysis of multiple responses, the software provided optimal enzymatic activity levels under the following conditions: 0.88 g L^−1^ *Ex*, inoculated with 2 × 10^7^ conidia mL^−1^ and pH 4. Based on the statistical significance obtained from the ANOVA, the regression models were constructed considering the significant linear, quadratic, and interaction terms. The model included the linear and quadratic terms of nitrogen concentration for chitinase activity. For β‐1,3‐glucanase activity, the model incorporated the linear and quadratic terms of nitrogen concentration and the interactions between nitrogen source and inoculum, nitrogen source and pH, and inoculum and pH. For protease activity, the model included the linear and quadratic terms of nitrogen concentration, the interaction between nitrogen source and inoculum, and the quadratic effects of inoculum concentration and pH. The general forms of the models are presented below:

$$\text{Chitinase Activity}=f\left(A,A^{2}\right).$$

$$\beta -1,3-\text{glucanase Activity}=f\left(A,A^{2},\mathrm{AB},\mathrm{AC},\mathrm{BC}\right).$$

$$\text{Protease Activity}=f\left(A,A^{2},\mathrm{AB},B^{2},C^{2}\right).$$

where A represents the nitrogen source concentration, B represents the initial inoculum concentration, and C represents the initial pH of the culture medium.

**TABLE 3:** ANOVA for Chitinase activity based on the Box–Behnken design.

Source	Sum of squares	DF	Mean square	*F*‐Value	*P*‐Value
A: Nitrogen Source	1786.53	1	1786.53	145.89	**0.0003**
B: Inoculum	61.9384	1	61.9384	5.06	0.0877
C: pH	0.227812	1	0.227812	0.02	0.8981
AA	1960.05	1	1960.05	160.06	**0.0002**
AB	26.3169	1	26.3169	2.15	0.2165
AC	7.48022	1	7.48022	0.61	0.4781
BB	0.41316	1	0.41316	0.03	0.8617
BC	19.4481	1	19.4481	1.57	0.2761
CC	81.6773	1	81.6773	6.67	0.1022
Lack of fit	83.2365	3	27.7455	2.27	**0.2229**
Pure error	48.9815	4	12.2454	—	—
Total (corr.)	4046.12	16	—	—	—

*Note:* R^2^ = 96.73%. Lack of Fit not significant (*p* > 0.05).

**TABLE 4:** ANOVA for β‐1,3‐glucanase activity based on the Box–Behnken design.

Source	Sum of squares	DF	Mean square	*F*‐Value	*P*‐Value
A: Nitrogen Source	103,294	1	103,294	15.01	**0.0061**
B: Inoculum	26650.5	1	26650.5	3.87	0.0898
C: pH	23349.6	1	23349.6	3.39	0.1081
AA	227,439	1	227,439	33.04	**0.0007**
AB	92641.1	1	92641.1	13.47	**0.0080**
AC	39728.5	1	39728.5	5.77	**0.0473**
BB	63486.3	1	63486.3	9.23	**0.0173**
BC	55885.0	1	55885.0	8.13	**0.0247**
CC	11324.0	1	11324.0	1.65	0.2405
Lack of fit	79089.3	3	26363.1	433.46	**0.0970**
Pure error	48187.6	7	6883.94	—	—
Total (corr.)	642,680	16	—	—	—

*Note:* R^2^ = 92.50%. Lack of Fit not significant (*p* > 0.05).

**TABLE 5:** ANOVA for Protease activity based on the Box–Behnken design.

Source	Sum of squares	DF	Mean square	*F*‐Value	*P*‐Value
A: Nitrogen Source	192,634	1	192,634	371.50	**0.0000**
B: Inoculum	606.739	1	606.739	1.17	0.3402
C: pH	207.163	1	207.163	0.40	0.5617
AA	415,305	1	415,305	800.92	**0.0000**
AB	4410.95	1	4410.95	8.51	**0.0434**
AC	516.426	1	516.426	1.00	0.3741
BB	5896.99	1	5896.99	11.35	**0.0275**
BC	905.408	1	905.408	1.75	0.2659
CC	5994.07	1	5994.07	11.56	**0.0253**
Lack of fit	6655.85	3	2218.62	4.28	**0.0970**
Pure error	2074.15	4	518.537	—	—
Total (corr.)	637,113	16	—	—	—

*Note:* R^2^ = 98.63%. Lack of Fit not significant (*p* > 0.05).

Data from experimental runs of RSM showed a rise in chitinase, β‐1.3‐glucanase, and protease activity levels (Table 6).

**TABLE 6:** Summary of Increased Enzyme Activity.

	Initial value^a^	Predicted value^b^	Real value^c^	Enzyme activity increase (%)^d^
Quitinase	28.95 U L^−1^	75.61 U L^−1^	74.91 U L^−1^	258%
β‐1,3 glucanase	591 U L^−1^	1450 U L^−1^	1520 U L^−1^	251%
Protease	391 mg L^−1^	971 mg L^−1^	967 mg L^−1^	247%

^a^ Maximum enzymatic activity observed during the initial selection of carbon and nitrogen sources.

^b^ Values predicted by the multiple response optimisation model.

^c^ Values obtained from the validation of the multiple response optimization model.

^d^ Percentage increase in enzymatic activity after the optimisation process.

The prediction of the multiple response optimisation model was validated with six experimental runs. The obtained results were 74.91 U L^−1^, 1510 U L^−1^, and 967 mg L^−1^ of chitinase, β‐1,3‐glucanase, and protease, respectively (Table 6). These results were in agreement with the predicted values.

### 3.5 Bio‐Control Experiment

The effect of the optimised enzymatic formulation as biocontrol on the germination of lettuce ( *L. sativa* ), a positive control of the phytopathogen *Fusarium* sp. present in the substrate, was observed. The treatments with the optimised enzymatic formulation and those with the *T. koningiopsis* conidia suspension showed statistically significant differences (*p* ≤ 0.05) compared to the absolute control and sick control treatments. The optimised enzymatic formulation was the treatment with the highest germination percentage, reaching 86.66%, compared to the 66.66% obtained with the conidia suspension. In contrast, the absolute and sick control treatments showed 36.66% and 13.33% germination percentages, respectively (Table 7).

**TABLE 7:** Effect of different treatments on lettuce seed germination and seedling development in the presence of *Fusarium* sp.

Treatments	Germinated seeds		Plant experiment					
	Media	Germination %	H^1^ (cm)	FBAP^2^ (g)	DBAP^3^ (g)	FRB^4^ (g)	DRB^5^ (g)	DSI^6^ %
(T1) Enzymatic formulation	17.33 ± 1.52^d^	86.66^d^	13.89 ± 2.02^c^	3.51 ± 0.95^c^	0.24 ± 0.14^b^	0.62 ± 0.71^b^	0.04 ± 0.03^b^	10
(T2) Suspension of conidia	13.33 ± 2.08^c^	66.66^c^	12,39 ± 1.78^bc^	3.03 ± 1.11^b^	0.16 ± 0.22^b^	0.29 ± 0.60^ab^	0.02 ± 0.02^ab^	15
(T3) Absolute Control	7.33 ± 2.51^b^	36.66^b^	10.82 ± 1.62^a^	1.58 ± 0.69^a^	0.08 ± 0.18^ab^	0.05 ± 0.07^a^	0.005 ± 0.003^a^	30
(T4) Sick Control	2.66 ± 2.08^a^	13.33^a^	8.60 ± 1.24^ab^	0.17 ± 0.39^a^	0.03 ± 0.069^a^	0.01 ± 0.021^a^	0.001 ± 0.001^a^	60

*Note:* Values are expressed as mean ± standard deviation. Different lowercase letters within the same column indicate statistically significant differences according to Tukey's test (*p* ≤ 0.05).

^1^ H: plant height (cm).

^2^ FBAP: fresh biomass of the aerial part (g).

^3^ DBAP: dry biomass of the aerial part (g).

^4^ FRB: fresh root biomass (g).

^5^ DRB: dry root biomass (g).

^6^ DSI: disease severity index (%).

**FIGURE 5:**
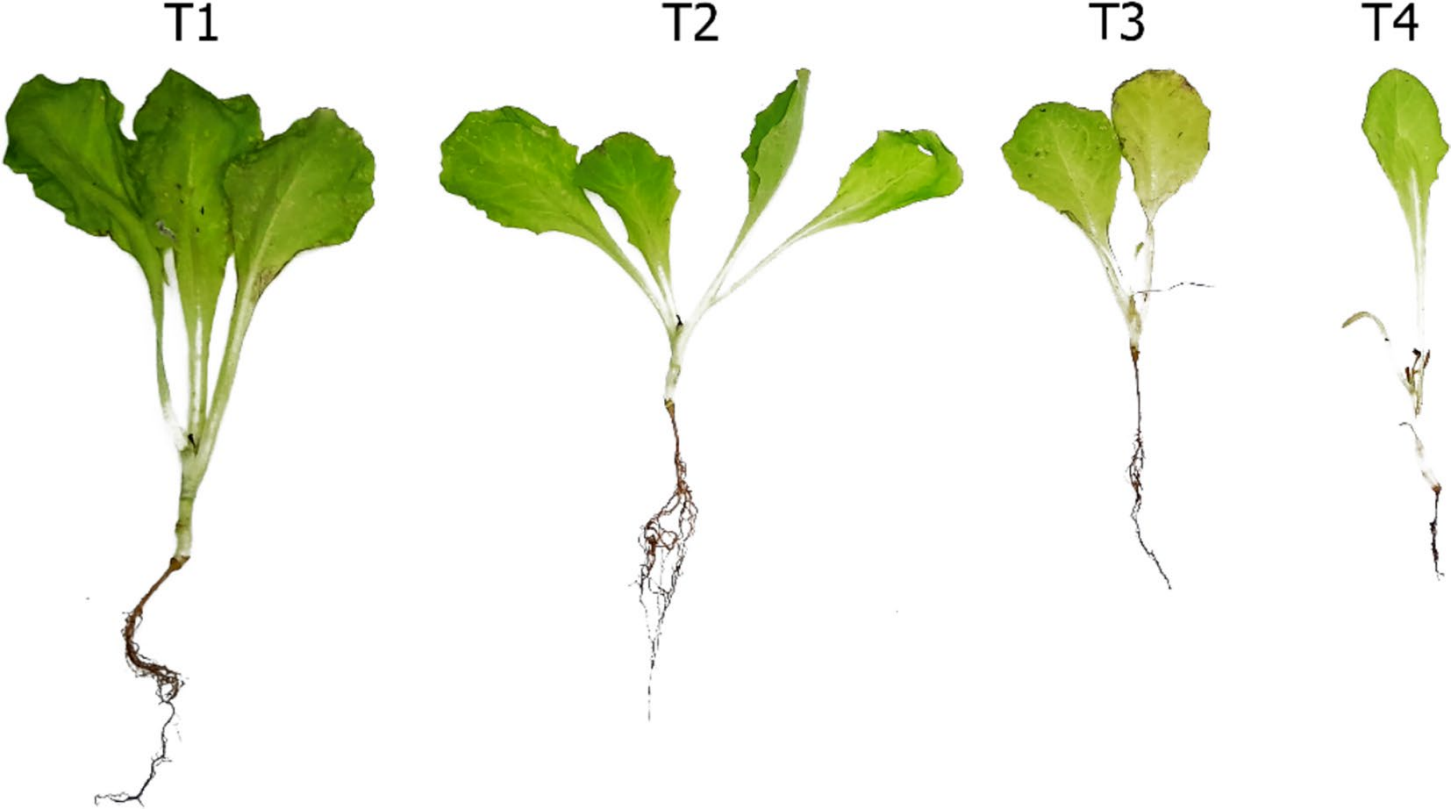
Lettuce seedlings after 30 days under different treatments. From left to right: (T1) Enzymatic formulation, (T2) Conidial suspension, (T3) Absolute control, (T4) Diseased control.

In the evaluation of biocontrol on lettuce seedlings, applying the optimised enzymatic formulation significantly enhanced plant development compared to the control treatments (*p* ≤ 0.05). As shown in Table 7 and Figure 5, seedlings treated with the enzymatic formulation exhibited the most significant overall growth, with a mean height of 13.89 ± 2.02 cm, fresh aerial biomass of 3.51 ± 0.95 g, and dry aerial biomass of 0.24 ± 0.14 g. These plants also showed significantly higher root biomass and lower disease severity index (DSI = 10%). The conidial suspension of LBM116 also improved plant development, although to a lesser extent, with a height of 12.39 ± 1.78 cm and DSI of 15%. In contrast, the absolute and diseased control seedlings exhibited reduced growth and higher disease symptoms, with 30% and 60% DSI values, respectively. The diseased control group showed stunted growth, chlorosis, and necrosis symptoms, as observed in Figure 5.

These findings demonstrate that the enzymatic formulation acts through two complementary mechanisms: suppressing disease development and stimulating plant growth.

## 4 Discussion

The agricultural sector has the inevitable challenge of maintaining crop production and profitability by replacing agrochemicals with agronomy based on more natural and sustainable practices. Using bioformulations on beneficial microorganisms or a combination of some of their extracellular lytic enzymes can help protect crops and conserve natural resources.

We have focused on the production of chitinase, β‐1,3‐glucanase, and protease by the *T. koningiopsis* LBM116 strain because it is a native strain adapted to the edaphoclimatic conditions of the study area, which favours its application in the field (Castrillo et al. 2021). Unlike commercial strains, very few studies have focused on optimising the enzymatic activity of native biocontrol strains. Therefore, we prioritised searching in nature, where a wide range of microorganisms and biocontrol strategies exist and can be used to manage pests and plant diseases in an environmentally sustainable way. Successful enzyme secretion by filamentous fungi requires analysing and optimising factors such as carbon and nitrogen sources, inoculum concentration, and physical variables like pH (Izarra et al. 2010; Dixit and Shukla 2023; Fellah et al. 2023).

In this work, we first studied the effect of carbon and nitrogen sources on the secretion of chitinase, β‐1,3‐glucanase, and protease enzymes in liquid medium by *T. koningiopsis* LBM116. The cell walls inactivated from phytopathogenic fungi presented the best results as carbon sources for the induction of enzyme secretion into the extracellular medium. Similar data have been reported by Ridout et al. (1986), who evaluated the cell walls of *Rhizoctonia solani*, and by Rao et al. (2016), who studied the cell walls of *Sclerotium rolfsii*.

In our study, similar to what was reported by Siddiquee (2017), an enhancement in enzymatic activity was observed with the rise in the concentrations of *Fusarium* sp. cell walls up to a certain level, after which the activity slightly decreased. The chitinase, β‐1,3‐glucanase, and protease enzyme activities were significantly higher at 7 g L^−1^ of *Fit I*. Our findings are supported by Ulhoa and Peberdy (1991), who observed a direct correlation between the concentration of cell walls and enzyme secretion up to a certain level. A decrease in enzyme activity beyond a specific concentration of cell walls may be the result of the accumulation of intermediate compounds resulting from the degradation of chitin, glucan, and protein in the medium, leading to the accumulation of a synthetic inhibitor of chitinase, β‐1,3‐glucanase, and protease themselves (Aida et al. 2014). The decrease in enzymatic activity after the increase in the concentration of carbon sources could also be due to genes linked to the catabolic repression of carbon, which are activated at high concentrations of carbon in the culture medium (Siddiquee 2017; Ulhoa and Peberdy 1991; Aida et al. 2014; Lorito et al. 1996).

Also, the enzymatic secretion of *T. koningiopsis* LBM116 was improved by optimising the nitrogen source of the culture media. The enzymatic activity was significantly higher with about 1 g L^−1^ of *Ex* as a nitrogen source. Gueye et al. (2020) have also reported *Ex* as a good inducer of enzymatic activity when used as a nitrogen source. All these results agreed with what was stated by Subramaniyan et al. (2001), that the Ex provides the fungus with amino acids and vitamins for its growth and induces extracellular enzymes such as chitinase, β‐1,3‐glucanase, and protease. But when the concentration of the *Ex* was increased to maximum levels, we observed a decrease in the enzymatic activity, which could be due to genes linked to the catabolic repression of nitrogen, which is activated at high concentrations of nitrogen in the culture medium (Olmedo‐Monfil et al. 2002).

In addition, chitinase, β‐1,3‐glucanase, and protease secretion by *T. koningiopsis* LBM116 were improved by optimising the inoculum concentration and physical variables (pH) in media containing *Fit I* and *Ex*. In this study, 1.2 × 10^7^ and 2 × 10^7^ conidia mL^−1^ similarly influenced enzymatic activity. We selected 2 × 10^7^ conidia mL^−1^ because it was the optimal prediction in the statistical model. The wide variety of types of assays in liquid medium made it impossible to truly compare the effective inoculum concentration for the induction of enzymatic activity among other fungi. It is still relevant, however, that this work allowed us to determine the inoculum concentration q that maximises the enzymatic secretion of *T. koningiopsis* LBM116.

About the optimization of the pH of the culture medium, the optimal pH was 4. This result coincides with the findings reported by Sandhya et al. (2004), who found maximum enzymatic production at pH 4 using *Trichoderma harzianum* in submerged fermentation. Also, the increase in the enzymatic secretion at acidic pH in the present study was in coincidence with the results of El‐Katatny et al. (El‐Katatny et al. 2000) with *T. harzianum*, and Gueye et al. (2020) with *Trichoderma asperellum*. However, a significant variation of pH ranges has been reported in favour of the secretion of chitinase, β‐1,3‐glucanase, and protease (Rao et al. 2016; Siddiquee 2017; Ulhoa and Peberdy 1991). These results suggest that the secretion of chitinase, β‐1,3‐glucanase, and protease may be co‐ordinately regulated, as all three enzymes were influenced in the same way by similar alterations of growth parameters in the culture media.

The bioassay conducted under controlled conditions showed that application of the optimised enzyme formulation derived from *T. koningiopsis* LBM116 improved seed germination (86.66%) and seedling development in the presence of *Fusarium* sp. Although a direct pathogen suppression assay was not performed, the improved plant performance under phytopathogenic stress conditions suggests an indirect biocontrol effect, likely mediated by increased physiological resilience. According to Harman et al. (1993), early colonisation by beneficial microorganisms during the first 12–24 h after application is crucial for establishing protective effects. Furthermore, the greater vigour observed in treated seedlings, reflected in greater aboveground and root biomass and a reduced disease severity index (DSI), supports the idea that enhanced plant growth may constitute a competitive advantage against pathogens. This dual function of growth promotion and disease mitigation is consistent with the findings of Innocenti et al. (2015), who reported that *Trichoderma* spp. not only induces resistance but also increases biomass even under water stress conditions. Pinto et al. (2014) also highlighted that more vigorous vegetative development is a key factor in reducing disease susceptibility. In this context, the dual function of the enzyme formulation—stimulating plant growth and reducing disease symptoms—positions it as a promising tool for sustainable agriculture. It contributes not only to plant health and early establishment but also to the long‐term resilience of crops under biotic stress, offering an alternative to conventional chemical control strategies.

## Author Contributions

Natalia Soledad Amerio and Marcela Paola Barengo designed the research and conducted the experiments. María Lorena Castrillo and Gustavo Angel Bich assisted in data analysis. Pedro Dario Zapata and Laura Lidia Villalba contributed to the manuscript writing. All authors contributed to the final version of the manuscript.

## Ethics Statement

The authors confirm that they have adhered to the ethical policies set by the journal.

## Conflicts of Interest

The authors declare no conflicts of interest.

## Data Availability

The data that support the findings of this study are available on request from the corresponding author. The data are not publicly available due to privacy or ethical restrictions.
